# HAS1^high^ cancer associated fibroblasts located at the tumor invasion front zone promote oral squamous cell carcinoma invasion via ECM remodeling

**DOI:** 10.1186/s13046-025-03493-6

**Published:** 2025-08-14

**Authors:** Wanyong Jin, Qiuya Yu, Liyuan Yu, Ting Zhou, Xiren Wang, Wanqiu Lu, Xiaoxin Zhang, Liang Ding, Qingang Hu, Yanhong Ni

**Affiliations:** 1 Central Laboratory of Stomatology, Nanjing Stomatological Hospital, Affiliated Hospital of Medical School, Institute of Stomatology, Nanjing University, No. 30 Zhongyang Road, Nanjing, 210008 China; 2 School of Biopharmacy, China Pharmaceutical University, Nanjing, Jiangsu China

**Keywords:** Oral squamous cell carcinoma, Cancer associated fibroblast, Invasion front, Extracellular matrix remodeling

## Abstract

**Background:**

Although tumor cell heterogeneity between the tumor center (TC) and invasion front (IF) of oral squamous cell carcinoma (OSCC) is well documented, the morphological, molecular, and functional characteristics of cancer-associated fibroblasts (CAFs) in these regions remain poorly understood.

**Methods:**

We examined hematoxylin and eosin (H&E)–stained OSCC sections to assess CAF morphology and correlation with patient prognosis. We then isolated paired CAFs from the tumor center (CAF^TC^) and invasion front (CAF^IF^) of four OSCC patients and compared their ECM-remodeling activity and pro-tumorigenic effects on OSCC cells. Furthermore, RNA sequencing identified differentially expressed genes between CAF^TC^ and CAF^IF^. Finally, based on RNA-seq findings, we knocked down hyaluronan synthase 1 (HAS1) in CAF^IF^ to evaluate its role in extracellular matrix (ECM) remodeling and tumor invasion.

**Results:**

Compared to CAF^TC^, CAF^IF^ exhibited a plump cell morphology and were associated with shorter disease-free survival. Functionally, CAF^IF^ showed higher ECM-remodeling activity and more effective ability for promoting OSCC invasion and lymph node metastasis than CAF^TC^. RNA-seq identified HAS1 was significantly upregulated in CAF^IF^, promoting hyaluronic acid (HA) production and ECM remodeling. HAS1 knockdown in CAF^IF^ diminished ECM remodeling and attenuated the ability of CAF^IF^ to promoting OSCC invasion.

**Conclusion:**

CAF^IF^ with plump cell morphology showed pro-invasive abilities, driven in part by HAS1 overexpression and ECM remodeling, suggesting that targeting HAS1-driven ECM remodeling could be a promising therapeutic strategy.

**Supplementary Information:**

The online version contains supplementary material available at 10.1186/s13046-025-03493-6.

## Background

Oral cancer is a worldwide prevalent malignant neoplasm, with approximately 389,000 new cases and 188,000 mortalities reported worldwide in 2022[1]. It is estimated that the number of new oral cancer cases will reach 642,000 by 2050[1]. Oral squamous cell carcinoma (OSCC) accounts for approximately 90% of oral cancer cases. The primary treatments for OSCC involve surgery, combinations with radiotherapy, chemotherapy, and targeted therapy. In clinical practice, the prognosis of patients is evaluated using the Tumor-Node-Metastasis (TNM) staging system, which includes three leading indicators: tumor size (T), cervical lymph node metastasis (N), and distant metastasis (M). The five-year survival rate is markedly lower in advanced-stage patients [2]. Understanding the mechanisms driving the development and progression of OSCC is essential for advancing adjuvant therapies to improve the prognosis of patients with advanced stage. However, current research on OSCC predominantly focuses on the tumor center (TC) while relatively little attention has been given to the invasion front (IF).

The IF of OSCC represents the leading edge of tumor invasion, typically around 1 mm in width. The molecular and morphological characteristics between the IF and the TC display differences. The tumor-to-stroma ratio at the IF is significantly lower [3], suggesting that tumor cells exhibit a more dispersed and aggressive phenotype in this region. Moreover, tumor cells at the IF show enhanced proliferative potential, with Ki-67 expression significantly higher at the IF than at the TC, and this expression correlates with clinical stage, tumor thickness, and lymph node metastasis [4]. Tumor cells of OSCC at the IF increase its invasion by downregulating Ras-Related Associated with Diabetes expression, which boosts metabolic activity [5]. These malignant features are not exclusive to OSCC, as similar traits are observed in other malignancies such as glioblastoma, breast cancer, and colon cancer[6–8]. For example, glioblastoma cells at the IF exhibit more significant invasion compared to those in the central region [6], and breast cancer cells at the IF also display increased proliferation and invasion [7]. These findings indicate that the IF is the most aggressive region of tumor tissues in tumor progression, and decoding this area is critical for understanding tumor progression. It also opens new avenues for precision and targeted therapies. Therefore, the cells composition and the molecular characteristics should be clarified.

The cancer-associated fibroblasts (CAFs) in OSCC and other malignancies have garnered increasing attention. CAFs are thought to influence tumor progression primarily by producing extracellular matrix (ECM) components and cytokines. Research has shown that CAFs promote tumor cell proliferation and migration by modifying ECM components, such as collagen[9]. Furthermore, CAFs secrete cytokines like TGF-β, which activate tumor cell signaling pathways, thereby enhancing tumor cell invasion [10]. Recently, the roles of CAFs at the invasion front (CAF^IF^) have become more attractive than CAFs at the tumor center (CAF^TC^), as they are complex because of their interaction with both tumor and immune cells. For instance, CAF^IF^ enhance tumor cell proliferation and promote epithelial-mesenchymal transition (EMT) via O-glycosylation of the CDK4-pRB axis[11]. CAFs in this region also significantly influence tumor invasion and metastatic potential through ECM remodeling, fibrosis, and collagen synthesis[12]. In esophageal squamous cell carcinoma, patients with high levels of a-SMA^+^ CAFs at the CAF^IF^ have a poorer prognosis, with aSMA^+^CAFs interacting spatially with CD163^+^ macrophages to suppress anti-tumor immune regulation[13]. Therefore, targeting CAF^IF^, particularly in ECM remodeling and cytokine regulation, represents a promising strategy to enhance OSCC treatment outcomes.

Extensive single-cell RNA sequencing (scRNA-seq) analyses have highlighted the heterogeneity and functional complexity of CAFs subtypes across various tumor types, mouse models, and human patients. For example, the inflammatory CAF (iCAF) subtype, are characterized by the secretion of cytokines and chemokines; the myofibroblastic CAF (myCAF) subtype, are involved in the deposition, modification, and degradation of the ECM; and the antigen-presenting CAF (apCAF) subtype, expresses MHC class II molecules, are all correlated with tumor progression with different functions. Hyaluronan synthase 1 (HAS1) is a key enzyme regulating the synthesis of hyaluronic acid (HA), a critical ECM component[14]; however, HAS1 is also a marker of iCAF subtype. Additionally, HAS1 has been shown to be upregulated in several inflammation-related diseases, including osteoarthritis, rheumatoid arthritis, and atherosclerosis [15–17]. However, the mechanism by which the upregulation of HAS1 expression in CAFs promotes invasion in OSCC remains unclear.

This study demonstrates that CAF^IF^ of OSCC have more significant clinical relevance and display stronger functional roles in promoting OSCC progression compared to CAF^TC^. CAF^IF^ enhance OSCC invasion by ECM remodeling. This mechanism suggests that targeting HAS1-mediated pathways could serve as a potential therapeutic strategy for OSCC.

## Method and materials

### Human subjects

All patients in this study were diagnosed with primary OSCC by experienced pathologists at the Pathology Department of Nanjing Stomatological Hospital, following hematoxylin–eosin (H&E) staining of tumor biopsy samples. OSCC tissues were classified and staged according to the World Health Organization (WHO) classification system and the TNM staging system by the Union for International Cancer Controlr (UICC). The patients included in this study received treatment at the Department of Oral and Maxillofacial Surgery of Nanjing Stomatological Hospital between 2015 and 2018. Inclusion criteria included histologically confirmed primary OSCC, accurate clinical parameters, curative intent surgery with negative margins, and follow-up periods exceeding 60 months, with death records up to March 1, 2024, the censor date. Exclusion criteria included patients diagnosed with T4b stage cancer, distant metastasis, a history of other head and neck malignancies, autoimmune diseases, or other malignancies, as well as pregnant or lactating individuals, due to potential physiological abnormalities that could affect tumor progression or treatment outcomes.

The histological classification of CAFs in OSCC was performed through a detailed pathological review of all tumor-containing formalin-fixed, paraffin-embedded blocks from 149 OSCC cases. Based on established histological criteria in the CAFs literature, specific and reproducible features were identified within OSCC tissues. Two distinct CAFs subtypes were characterized: Thinner morphology CAFs, defined by their thin cellular morphology and organized arrangement, and plumper morphology CAFs, defined by a plump morphology and a more chaotic arrangement.

### Definition of the IF and TC

In this study, the IF was defined as a 1 mm-wide region extending inward from the tumor–stroma interface at the boundary between tumor tissue and adjacent normal tissue, based on previously established criteria[18]. The TC was defined as the remaining internal region of the tumor, distant from the invasive margin. Digital slide images of H&E-stained sections were used for regional annotation. Under 40 × magnification, the tumor boundary was first identified and marked using digital pathology software. A contour 1 mm inside the boundary was then drawn to demarcate the IF. The area inside this contour was defined as the TC region. All annotations were independently reviewed by two trained pathologists, and any discrepancies were resolved by consensus.

### Cell culture

The HSC3 cell line was purchased from Merck (# SCC193), and the CAL27 cell line was obtained from ATCC (# CRL-2095). The HN6 cell line, originally derived from human tongue squamous cell carcinoma, was kindly provided by the Laboratory of Oncology Biology, Ninth People’s Hospital, Shanghai Jiao Tong University School of Medicine. All tumor cell lines were cultured in Dulbecco’s Modified Eagle Medium (DMEM, Invitrogen, #C11995500BT) supplemented with 10% fetal bovine serum (FBS) and 1% penicillin–streptomycin, and maintained in a humidified incubator at 37 °C with 5% CO₂. Cancer-associated fibroblasts were isolated from oral squamous cell carcinoma tissues collected during surgery and expanded in vitro using fibroblast medium (FM, ScienCell, #2301) supplemented with 2% FBS, 1% fibroblast growth supplement, and 1% penicillin–streptomycin. The OSCC and CAF cell lines were maintained under sterile conditions, and no mycoplasma contamination was observed during routine culture. The cell lines were maintained under standard conditions, and their identity was routinely monitored based on established characteristics, including short tandem repeat references.

### Mouse model

To generate an orthotopic xenograft tongue tumor model, 2 × 10^5^ HN6 OSCC cells were mixed with 1 × 10^6^ CAF cells and suspended in 20 μL of PBS/Matrigel (3:1). The mixture was injected into the anterior tongue of male BALB/c-nude mice (4–5 weeks old) using a 30-gauge syringe (BD Biosciences). After 3 weeks, the mice were euthanized by cervical dislocation, and their tongues and submandibular lymph nodes were harvested. All animal experiments were conducted according to the animal experimental protocol approved by the Animal Welfare and Ethics Review Committee of Nanjing University. The mice were maintained under the following conditions: a 12-h light/12-h dark cycle, a temperature of 72 °F (22 °C), and a humidity of 40–50%.

### Immunohistochemistry (IHC) and immunofluorescence experiments

All procedures were performed on 4 μm human or mouse tissue sections. Briefly, for OSCC samples or mouse tumor IHC staining, the slides were dewaxed and subjected to antigen retrieval (heat-induced). After blocking with 3% bovine serum albumin (BSA), the sections were incubated overnight at 4 °C with the primary antibodies (HAS1 1:200; CK 1:100; E-cadherin 1:200; N-cadherin 1:100). The unbound primary antibodies were washed off, and the slides were incubated with secondary antibodies at room temperature for 1 h. Then, staining was developed using a DAB staining kit. After staining, the slides were washed with tap water, counterstained with hematoxylin, dehydrated, and mounted. For cell immunofluorescence staining, the cells were fixed with 4% paraformaldehyde for 15 min, permeabilized with 100% methanol, and then blocked with 3% BSA. The cells were incubated overnight with the primary antibodies. After three washes with PBS, the coverslips were incubated with secondary antibodies conjugated to fluorescent dyes (anti-mouse/rabbit Alexa Fluor Plus 488/594, dilution: 1:400) at room temperature in the dark for 1–2 h, followed by DAPI staining (Bioword, China).

Quantitative analysis of IHC and immunofluorescence staining was performed by two independent pathologists, who were blinded to the clinical data. The median value was used as the cutoff for further analysis. The staining score for HAS1 was defined by the product of the staining percentage and intensity. Intensity was defined as follows: 1 = negative; 2 = weak; 3 = moderate; 4 = strong. Low/medium/high staining was defined as the 0-25th percentile, 25th-75th percentile, and 75th percentile-maximum value, respectively.

### Isolation and identification of CAF cell lines derived from OSCC patients

Primary CAFs cell lines were derived from OSCC tumor tissues[19]. Fresh biopsy tissue was obtained during surgery from the primary lesion of advanced OSCC patients and immediately stored in DMEM at 4 °C for 30–50 min for pathological analysis. H&E staining was performed on frozen samples during the surgery to ensure the samples contained the IF, TC, and normal areas. The samples were washed with PBS and antibiotics, and the epithelial and adipose tissues were removed. Under the guidance of a pathologist, a 16-g biopsy needle was used to collect tumor tissue 1 mm in width from both the IF and TC. The tissue was digested with an enzyme mixture consisting of 0.2% collagenase II/IV (#BS033A/035A, Biosharp), 0.08% neutral protease (#J10050, Shanghai BlueKey), and 0.0032% hyaluronidase (#H3506, Sigma) for 30 min. The remaining small tissues were placed in a cell culture flask containing fibroblast medium (FM) and incubated at 37 °C. The medium was replaced every 2–3 days. The remaining tissue was used to prepare H&E slides to confirm the tissue collected from the IF.

In the following days, other cell types were removed through gradient centrifugation, digestion, and repeated adherence methods. CAFs were immortalized by infection with hTERT to achieve stable long-term cultivation of the CAF cell lines. CAF cell lines derived from different patients were numbered according to the sampling time, marked as #9.27, #10.20, #11.14, and #12.13. To avoid cross-contamination, CAF cell lines from different patients were cultured separately in each independent experiment. The #9.27 and #10.20 CAF cell lines were selected for further experiments and validation, designated as CAF1 and CAF2, respectively.

### Cell proliferation assay

A total of 3000 cells per well were seeded into 96-well plates and cultured for 24, 48, and 72 h to observe the cell proliferation at different time points. At the specified time points (24, 48, 72 h), 10 μL of Cell Counting Kit-8 reagent (Dojindo, Japan) was added to each well. This reagent reflects cell proliferation activity by detecting cell metabolic products. The 96-well plates were incubated at 37 °C with 5% CO₂ for 2 h, an optimized incubation time to ensure the best sensitivity of the reaction. The optical density value was measured at 450 nm using the Varioskan Flash microplate reader.

### Cell migration assay

The cell migration assay was performed in Transwell chambers with an 8 μm pore size (Corning, New York, USA). 1 × 10^4^ cells were seeded into the upper chamber containing 100 μL of serum-free medium to ensure uniform cell distribution. The lower chamber was filled with a medium containing 10% fetal bovine serum as a chemoattractant. After 6 h of culture, cells that had migrated to the bottom of the membrane were fixed with methanol and stained with crystal violet. The results were determined by observing and counting the stained cells in five randomly selected fields under an optical microscope (10 × magnification).

### Gel contraction assay

To estimate the ECM remodeling capability mediated by CAFs, 1 × 10^6^ CAFs were embedded in a 0.2 ml mixture of collagen I/Matrigel (1:1) and seeded into the upper chamber of a 24-well Transwell plate (Corning, New York, USA, 8 μm). After gel solidification, fibroblast medium (#2301, Sciencell, USA) was added to the lower chamber, and the cultures were maintained at 37 °C with 5% CO₂. Acellular gels prepared using the same matrix composition without cell addition were included as cell-free controls. Gel contraction was monitored daily by imaging. The relative area of each well and gel was measured and analyzed using ImageJ software. To estimate the gel contraction, the percentage of contraction was calculated using the formula: 100 × (well area − gel area)/well area.

### Organoid culture system for cell growth and invasion assays

To form heterotypic organoids, 8 × 10^4^ GFP-labeled CAFs were cultured with 2 × 10^4^ mCherry-labeled HN6 cells or 8 × 10^4^ unlabeled CAFs with 2 × 10^4^ GFP-labeled HN6 cells. After 2 days of culture, stable heterotypic spheroids were embedded in a collagen I/Matrigel (3:1) mixture and continued to be cultured at 37 °C, 5% CO₂ for an additional 5 days. Invasion by CAF/HN6 heterotypic organoids was then determined. Invasion of the organoids was estimated using ImageJ software, with data represented by the maximum invasion distance (Lmax). Lmax is defined as the distance from the spheroid surface to the most distant invading cells. To measure the growth of CAF/HN6-derived heterotypic spheroids, 6 × 10^4^ HN6 cells were mixed with 6 × 10^4^ CAF cells and cultured in ultra-low attachment culture plates (S-bio, #ms9096vz). After 2 days, stable heterotypic spheroids were formed and collected after 10 days for organoid diameter analysis. The maximum area of the organoids was measured using ImageJ software.

### RNA analysis

RNA was extracted using Trizol reagent according to the manufacturer’s instructions, and cDNA was synthesized using HiScript III RT SuperMix (Nanjing VeroScience Biotechnology Co., Ltd). The relative expression of genes was quantified using AceQ® qPCR SYBR® Green Master Mix (Nanjing VeroScience Biotechnology Co., Ltd). All primers were synthesized by Nanjing Yijing Biotechnology Co., Ltd. The primer sequences are as follows:

- ELN: F: GCAGGAGTTAAGCCCAAGG / R: TGTAGGGCAGTCCATAGCCA
- MCAM: F: AGCTCCGCGTCTACAAAGC / R: CTACACAGGTAGCGACCTCC
- ICAM2: F: CGGATGAGAAGGTATTCGAGGT / R: CACCCACTTCAGGCTGGTTAC
- HAS1: F: GAGCCTCTTCGCGTACCTG / R: CCTCCTGGTAGGCGGAGAT
- ADGRG1: F: CCAGCGGAACCAGACACAC / R: TCTTCGGAGTTCTCGATGGAG
- NOG: F: CCATGCCGAGCGAGATCAAA / R: TCGGAAATGATGGGGTACTGG
- SCN5A: F: TCTCTATGGCAATCCACCCCA / R: GAGGACATACAAGGCGTTGGT
- LSP1: F: GGAGCACCAGAAATGTCAGCA / R: TCGGTCCTGTCGATGAGTTTG
- RIPK4: F: GATCTCCGGTTCCGAATCATC / R: TCAGAAATCTTGACGTGGTAGTG
- TMEM132B: F: GGGAGTGACAGAGAGTCGAGG / R: AGTTCGTGGGGAGGTAAGCA
- CDH1: F: GAACGCATTGCCACATACAC / R: GAGGATGGTGTAAGCGATGG
- CDH2: F: ATGGAAGGCAATCCCACTAA / R: CAGTAGGATCTCCGCCACTG
- VIM: F: GTACCGGAGACAGGTGCAGT / R: CTCAATGTCAAGGGCCATCT

### Western blotting

Cells were scraped from the culture and lysed on ice using RIPA lysis buffer (Beyotime, #P0013B) containing protease and phosphatase inhibitors to obtain the cell lysate. Equal amounts of protein were separated by SDS-PAGE and blocked with 3% BSA for 1 h at room temperature. After incubating with the primary antibody (HAS1 1:2000; E-cadherin 1:1000; N-cadherin 1:2000) overnight at 4 °C, the membrane was incubated with HRP-conjugated secondary antibody for 1 h. Protein bands were detected using a protein imaging system (Tanon 5200).

### Reagents

α-SMA (#67,735–1-lg), N-cadherin (#22,018–1-AP) and COL1A1 (#67,288–1-1 g) were obtained from Proteintech. HAS1 (#YM0324) was obtained from Immunoway. PAN-CK (#ab9377) was obtained from Abcam. E-cadherin (#3195) was obtained from Cell Signaling Technology. Secondary anti-mouse IgG Dylight 488 (#35,502) and anti-mouse IgG Dylight 594 (#35,510) were obtained from ThermoFisher.

### Enzyme-Linked Immunosorbent Assay (ELISA)

CAFs culture supernatants were collected, centrifuged (3000 rpm for 10 min) to remove cell debris, and the supernatant was stored at −80 °C to avoid repeated freeze–thaw cycles. The level of HA secreted by CAFs was detected using a commercial ELISA kit (Cusabio, #CSB-E04805h) according to the manufacturer’s protocol.

### Lentiviral transfection

To stably knock down HAS1, three shRNA-HAS1 constructs were designed and transfected into lenti-Cas9-Blast plasmids (GenePharma). The lentiviral particles were produced by transfecting 293 T cells, and the virus was concentrated using the Lenti-X Concentrator (Takara Bio Inc.). CAFs were infected with the virus and selected with 5 μg/mL puromycin for 2 weeks to establish stable cell lines.

### Transwell-based indirect co-culture model

To assess whether CAF^IF^ regulates the migratory ability of OSCC cells via secreted cytokines and chemokines, an indirect co-culture model was established using 24-well Transwell inserts with 8-μm pore membranes (Biofil, #TCS020024). CAF^IF^-NC or CAF^IF^-shHAS1 cells were first seeded into the lower chambers and allowed to adhere overnight. On the following day, HN6 cells were seeded into the upper inserts in serum-free DMEM. After 24 h of co-culture, non-migrated cells on the upper surface of the membrane were gently removed using a cotton swab. The cells that had migrated to the underside of the membrane were fixed with 4% paraformaldehyde, stained with crystal violet, and counted in five randomly selected fields under a microscope. The number of migrated HN6 cells was used to evaluate the effect of CAFs-derived cytokines and chemokines on OSCC cell motility in a non-contact setting.

### CAF-derived decellularized ECM model

CAF^IF^-NC and CAF^IF^-shHAS1 cells were seeded in 48-well plates and cultured until full confluence. Fibroblast medium was then applied and refreshed daily for 7 days, with gentle PBS rinses to promote extracellular matrix deposition. On day 7, cells were decellularized using 0.5% Triton X-100 and 20 mM NH₄OH for 2 min, followed by multiple PBS washes. The resulting matrix was retained as CAF-derived decellularized ECM (dECM)[20]. Tumor spheroids were generated using the hanging drop method (2 × 10^3^ cells per spheroid) and seeded onto the surfaces of CAF^IF^-NC or CAF^IF^-shHAS1 dECM. After a 30-min adhesion phase, serum-free DMEM was added, and spheroids were incubated for 24 h[21]. Spheroid invasion was assessed by fluorescence microscopy. The maximum invasion distance (Lmax) from the spheroid edge was measured using ImageJ software to compare the effects of different CAF-derived ECM on OSCC invasiveness. For EMT analysis, OSCC cells (HN6, CAL27, and HSC3) were cultured directly on the CAF-derived dECM for 24 h, after which RNA and protein were extracted for qPCR and Western blot detection of EMT-related markers[20].

### Statistics and reproducibility

The statistical tests used for each figure are indicated in the figure legends. All samples represent biological replicates. Data are expressed as mean ± SD. For both in vitro and in vivo analyses, experiments were independently repeated three times and twice, respectively, with similar results to demonstrate reproducibility. Statistical analysis was performed using GraphPad Prism 8 and SPSS software (SPSS Inc.). When appropriate, the Kolmogorov–Smirnov test was used to assess normality. The T-test, Mann–Whitney U test, ANOVA, and Kruskal–Wallis test were used to compare differences between two or more groups. Survival analysis, including overall survival (OS) and disease-free survival (DFS), was assessed using the Kaplan–Meier method and log-rank test. Cox proportional hazard regression models were used to determine independent prognostic significance by analyzing multivariable hazard ratios (HR) and 95% confidence intervals (CI). Individual *P*-values are shown when indicated.

## Results

### Prevalence of “Plumper morphology” CAFs is higher in the IF than in matched TC components

**Fig. 1:**
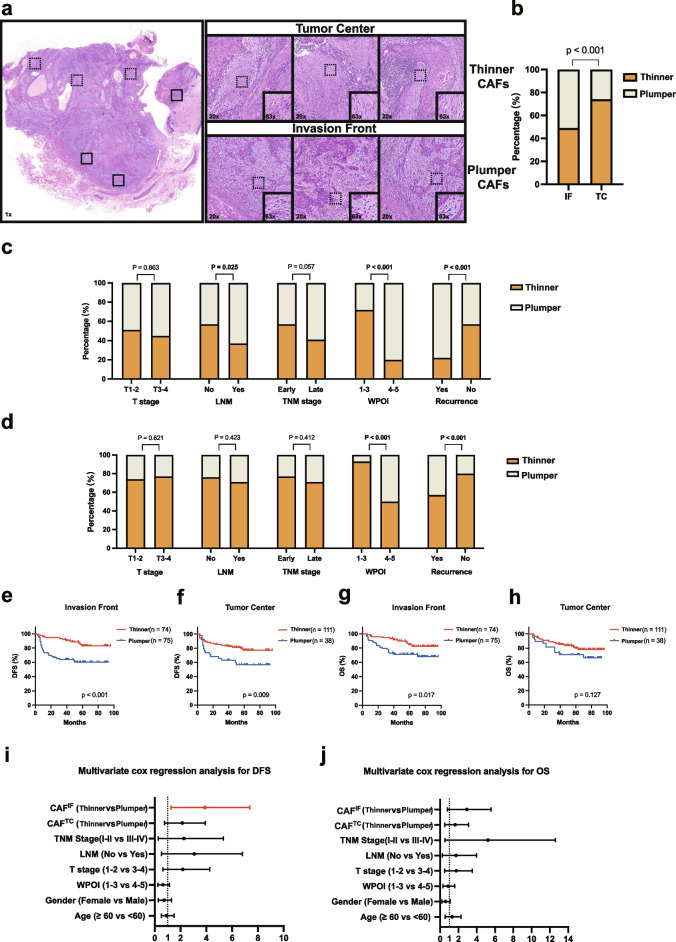
Superior Clinical Value of CAFs at the Invasion front. **a** Representative images of two types of CAFs in the tumor center and invasion front of OSCC patients stained with H&E. **b** The graphical depiction illustrates the distribution ratio of CAFs types in the tumor center and invasive front of 149 OSCC patients. *P*-values were calculated using Chi-squared test. **c**, **d** Bar graph showing the association between clinicopathological parameters of 149 OSCC patients and CAF types at the tumor center and invasive front. *P*-values were calculated using Chi-squared test. **e**–**h** Graphical depiction of OS and DFS in 149 OSCC patients, categorized by thinner CAFs and plumper CAFs. *P*-values were calculated using the Log-rank test. **i**, **j** Graphical depiction of the independent prognostic significance of tumor center and invasive front CAFs types in OS and DFS in 149 OSCC patients, as determined by multivariable analysis (Cox proportional hazards model)

To elucidate the differences between CAF^IF^ and CAF^TC^, we first classified the histopathological features of CAFs according to prior studies. Analysis of 149 OSCC H&E slides from our institution showed that there was a significant difference between these two regions (*p* < 0.001) (Fig. 1a-b), with plumper morphology CAFs predominating at the IF (51%) and thinner morphology CAFs more prevalent at the TC (74%).

Additionally, we examined the relationship between the morphologies of CAFs in the TC and IF with clinical-pathological parameters of OSCC. Results revealed that higher number of plumper morphology CAFs at IF were significantly associated with more lymph node metastasis (LNM) (*p* = 0.025), the poorer Worth pattern of invasion (WPOI) (*p* < 0.001), and higher postoperative recurrence rate (*p* < 0.001) (Fig. 1c). In contrast, higher number of plumper morphology CAFs at TC were only significantly correlated only with poorer WPOI (*p* < 0.001) and higher postoperative recurrence rate (*p* < 0.001) but not correlated with lymph node metastasis (Fig. 1d).

## “Plumper morphology” CAFs located in the IF is an independent prognostic factor for OSCC

To validate the prognostic value of CAF^IF^ and CAF^TC^ of OSCC, we assessed the ability of CAFs to differentiate OS and DFS among OSCC patients at the TC and the IF. The log-rank test indicated that patients with plumper CAFs at the IF had poorer DFS and OS compared to those with thinner CAFs (*p* < 0.001; *p* = 0.017). In contrast, within the TC, only plumper CAFs showed poorer DFS relative to thinner CAFs (*p* = 0.009), with no significant difference in OS (*p* = 0.127) (Fig. 1e-h).

Furthermore, using the COX regression model, we examined the independent prognostic importance of CAFs in both regions. The results indicated that CAF^IF^ served as independent prognostic indicators for OSCC patients concerning DFS, in contrast to CAF^TC^ (Fig. 1i-j). This discovery raises the possibility of phenotypic and functional variations between CAF^IF^ and CAF^TC^, underscoring their clinical significance. Consequently, exploring these differences in phenotype and function, as well as the underlying molecular mechanisms may make it easier to find novel therapeutic targets for OSCC.

## Pro-tumor abilities of CAF^IF^ components are significantly higher than matched CAF^TC^ components

**Fig. 2:**
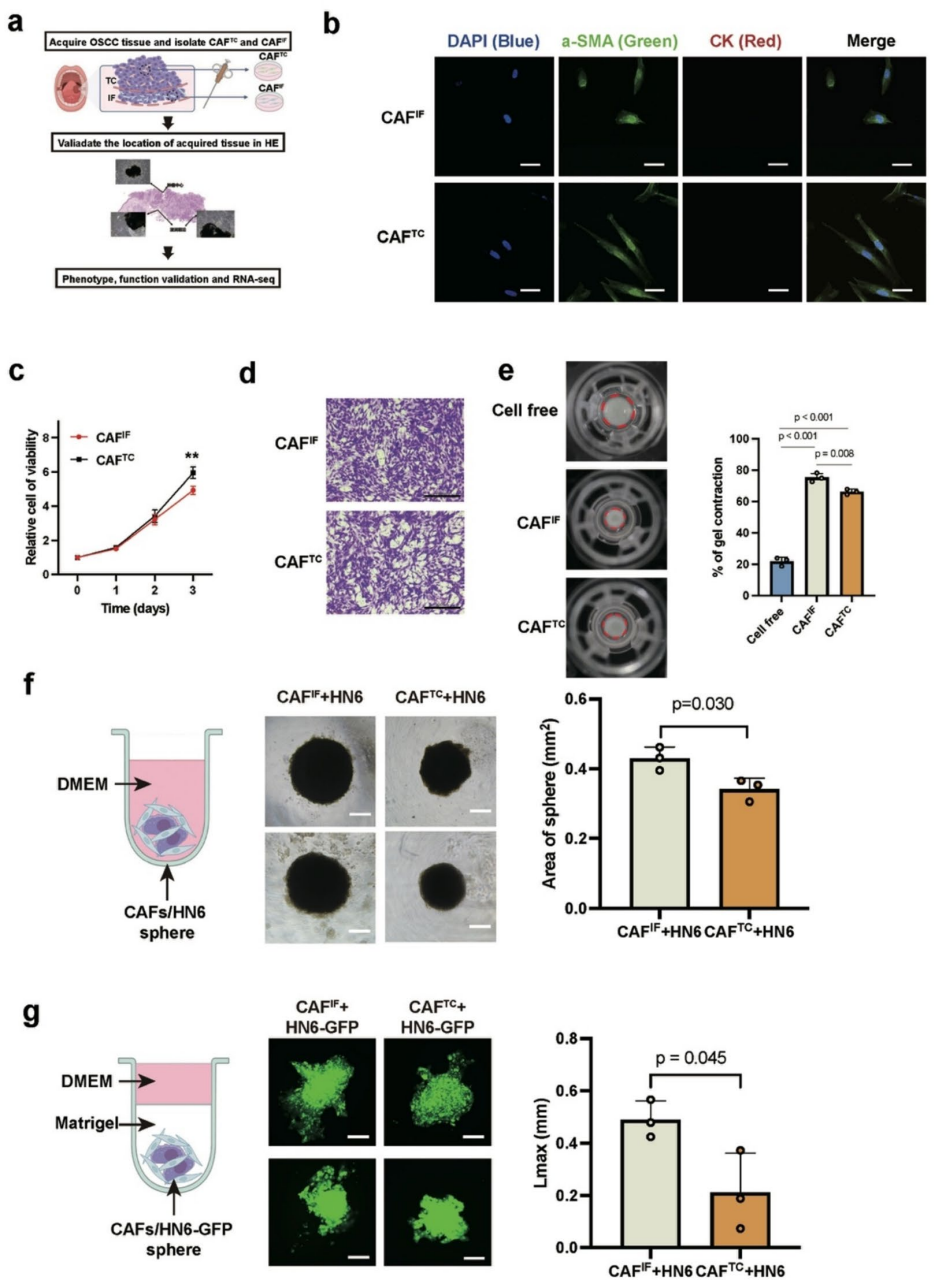
The CAFs at the invasion front promote the progression of OSCC. **a** Schematic of the protocol used to isolate, culture, and establish primary human CAF^IF^ and CAF^TC^ based on tumor center and invasion front from patients. **b** Immunofluorescence staining shows that CAFs from both tumor center and invasion front highly express α-SMA and do not express pan-CK. Scale bar, 20 μm. **c** The line graph shows the comparison of proliferation ability between CAF^IF^ and CAF^TC^ using CCK-8 assay. *P* = two-tailed t test. **d** The graph shows the comparison of migration between CAF^IF^ and CAF^TC^ using Transwell assays. Scale bar, 200 μm. **e** ECM remodeling was evaluated using a Matrix gel contraction assay (*n* = 3) among cell-free control, CAF^IF^, and CAF^TC^. *P* = two-tailed t test. **f** Co-culture of HN6 with CAF^IF^ or CAF^TC^ to form heterotypic spheroids and graphical representation of organoid diameter. *P* = two-tailed t test. Scale bar, 200 μm. **g** Setup of heterotypic organoid formation from HN6-GFP and CAF^IF^ or CAF^TC^, cultured in Matrigel gels mixed with collagen I. The matrix invasion of CAF/HN6 heterotypic organoid was determined by estimating the maximal distance of invasion from the spheroid border (Lmax). *n* = 3/group, *P* = two-tailed t test. Scale bar, 100 μm

To further investigate the phenotypic and functional differences between CAF^IF^ and CAF^TC^, we obtained paired primary CAF samples from these regions in four OSCC patients (#9.26, #10.20, #11.23, #12.13) using biopsy needles. H&E staining of pathological sections confirmed that the tissues were sampled from the TC and IF, respectively (Fig. 2a). Morphological observation under immunofluorescence revealed that CAF^IF^ exhibited a plumper cytoplasmic morphology with rounder and broader cell bodies, whereas CAF^TC^ displayed a thinner, elongated, spindle-shaped appearance (Fig. 2b). These differences were consistently observed in α-SMA-positive cells (Fig. 2b). Immunofluorescence analysis demonstrated high expression of the classic CAFs marker α-SMA, while lacking Cytokeratin (CK) expression, verifying that these cells were fibroblastic rather than epithelial, (Fig. 2b). For further analysis, primary CAFs were immortalized by infection with human telomerase reverse transcriptase (hTERT) lentiviral particles.

The CCK-8 assay revealed that CAF^TC^ exhibited a significantly higher proliferation rate compared to CAF^IF^ (*p* = 0.002) (Fig. 2c). However, migration assays showed no significant difference in migratory capacity between CAF^TC^ and CAF^IF^ (Fig. 2d). But matrix contraction assays indicated that CAF^IF^ possessed more potent matrix remodeling abilities compared to CAF^TC^ (*p* = 0.008, Fig. 2e).

To examine the mechanisms of CAF^TC^ and CAF^IF^ differentially influencing tumor proliferation and invasion, we co-cultured CAFs with HN6 cells in an in vitro organoid model. CAF^IF^ significantly enhanced HN6 cell proliferation compared to CAF^TC^, as measured by tumor sphere area (Fig. 2f). Embedding tumor spheres composed of CAFs and HN6-GFP cells in a Matrigel and collagen I mixture and observing under confocal microscopy, we found that the maximal distance of invasion from the spheroid border (Lmax) was significantly increased in HN6 cells with CAF^IF^ compared to those with CAF^TC^ (Fig. 2g).

**Fig. 3:**
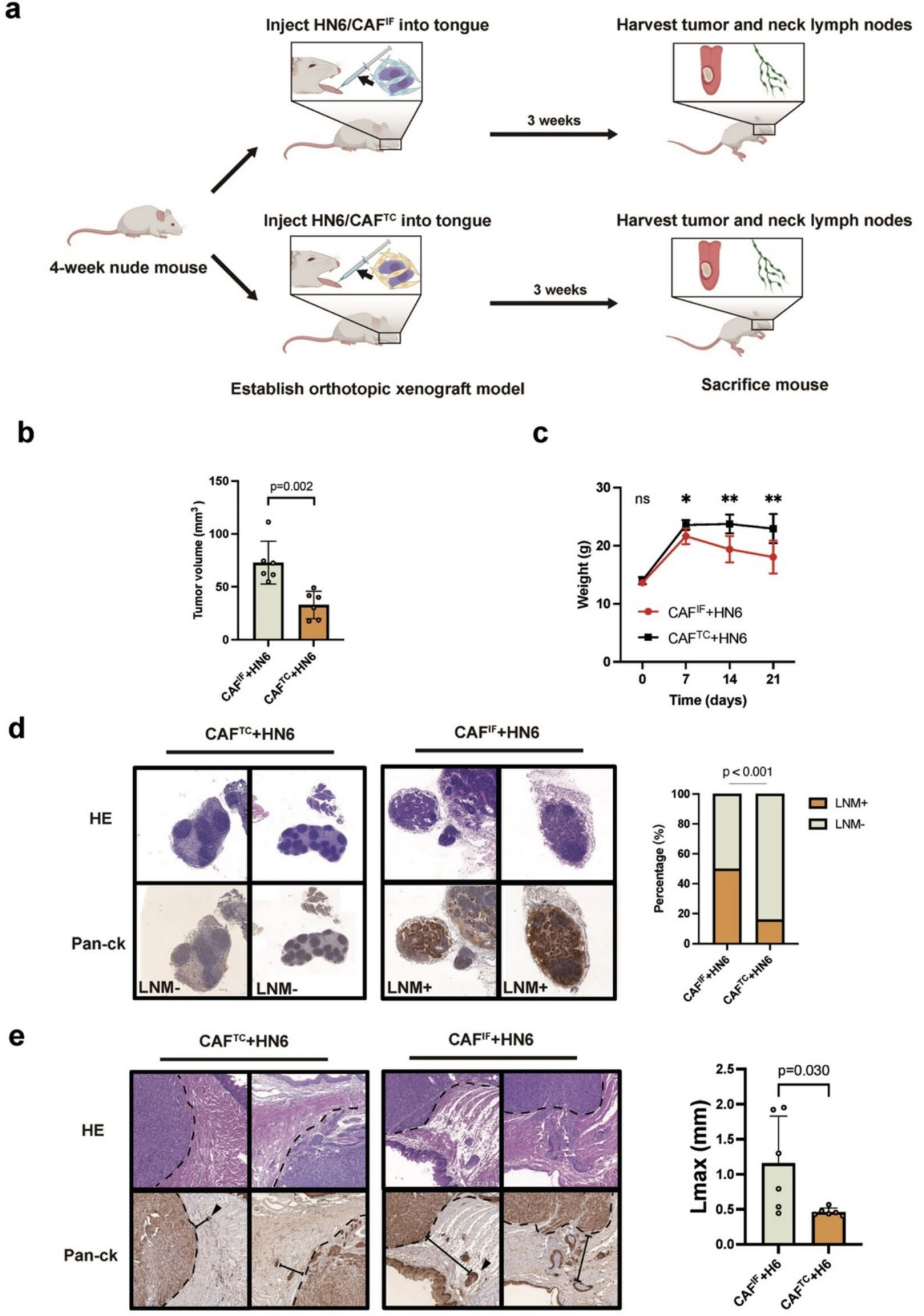
The CAF^IF^ promote the progression of OSCC in vivo. **a** Graphical representation of the construction of an orthotopic tongue tumor model in mice by co-injecting HN6 and CAF^IF^ or CAF.^TC^ cells into the anterior tongue. The mouse tongues and submandibular lymph nodes were collected three weeks later. **b** Graphical representation of tumor volume. Two-tailed t test (*n* = 6/group). **c** Graphical representation of changes in mouse weight following tumor implantation. Two-tailed t test (*n* = 6/group). **d** Graphical representation of LNM percentage. Chi-squared test (*n* = 6/group). Scale bar: 200 μm. **e** Graphical representation of invasion distance in satellite lesions. Two-tailed t test (*n* = 6/group)

For in vivo analysis, we established an orthotopic tongue tumor model in mice by injecting a mixture of CAFs and HN6 cells into the mouse tongue (Fig. 3a). Tumor volume comparison showed that CAF^IF^ enhanced tumor growth more significantly than CAF^TC^ (*p* = 0.002) (Fig. 3b). Additionally, analysis of submandibular lymph node metastasis indicated that CAF^IF^ promoted a higher metastatic potential (*p* < 0.001, Fig. 3d). CAF^IF^ also resulted in a greater maximum invasion distance in satellite lesions than CAF^TC^ (*p* = 0.030, Fig. 3e). Collectively, these results indicated that CAF^IF^ markedly promoted tumor cell proliferation, local invasion, and lymph node metastasis.

## CAF^IF^ are characterized by high HAS1 expression and HAS1^high^ CAFs predict poor prognosis of OSCC

**Fig. 4:**
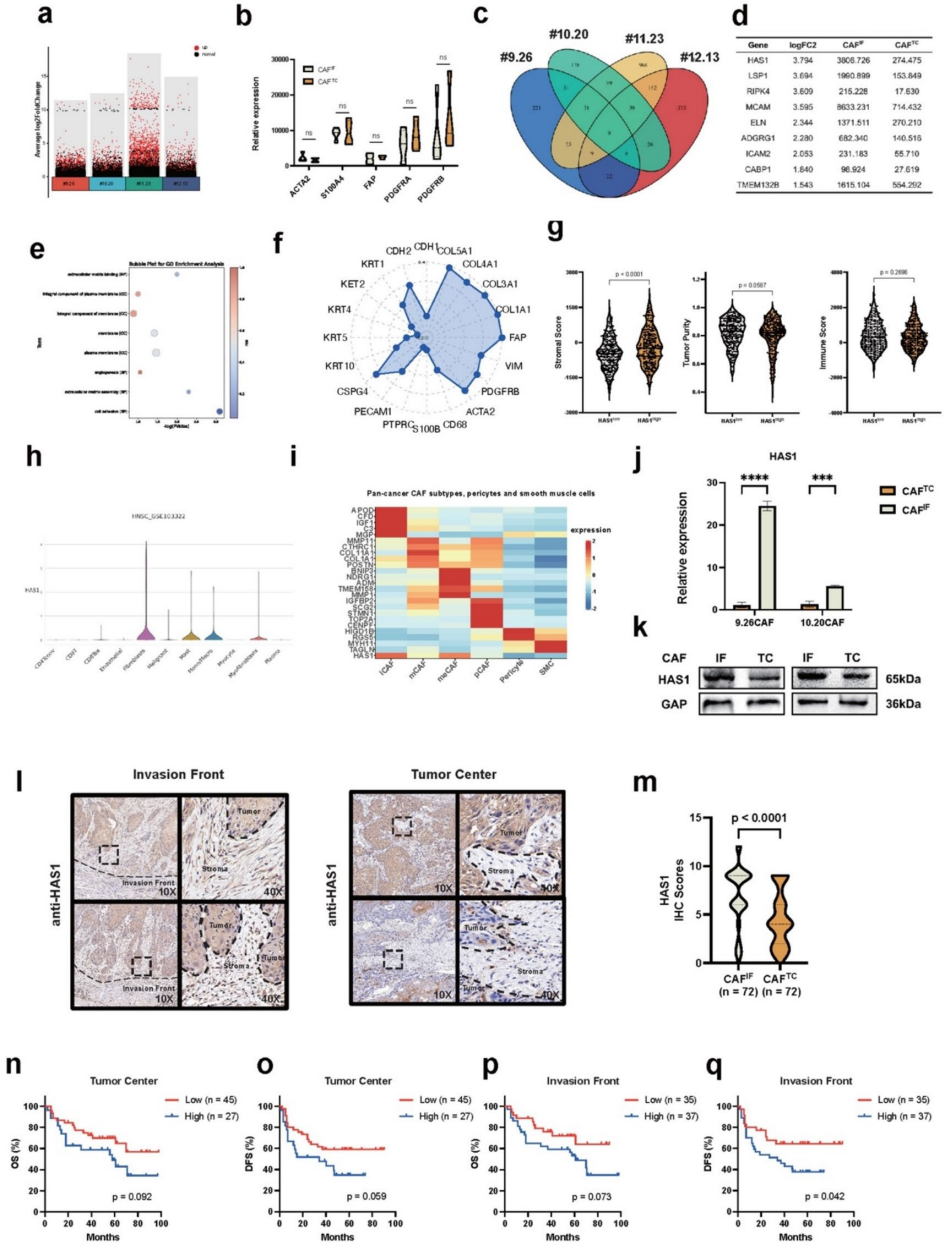
The CAF^IF^ is characterized by high HAS1 expression. **a** Graphical representation of genes highly expressed in CAF^IF^ compared to CAF^TC^, identified using the EB-Seq algorithm in #9.27, #10.20, #11.14, and #12.13 CAFs, under the following criteria: i) Fold Change > 2 or < 0.5; ii) FDR < 0.05. **b** Graphical representation of the relative expression of classical CAF markers in CAF^IF^ (*n* = 4) and CAF^TC^ (*n* = 4). Two-tailed t test. **c** Venn diagram showing the highly expressed genes in CAF^IF^ from #9.27, #10.20, #11.14, and #12.13. **d** The relative expression of up-regulated gene in CAF^IF^ from #9.27, #10.20, #11.14, and #12.13. **e** Representation of GO analysis from RNA sequencing and differential gene analysis of CAF^IF^ and CAF^TC^. **f** Correlative analysis between HAS1 gene expression and other stromal cell markers in TCGA-HNSC (*n* = 522) database. **g** The tumor purity, stromal score, and immune score of patients with high/low HAS1 expression were evaluated using the Estimate algorithm in the TCGA-HNSC (*n* = 522) database. **h** HAS1 expression in head and neck squamous cell carcinoma with Tisch2 (http://tisch.comp-genomics.org/home/). **i** Heatmap showing the expression of HAS1 and representative marker genes of distinct CAF subtypes (iCAF, mCAF, meCAF, pCAF), pericytes, and smooth muscle cells, based on data from the PanCAF dataset (https://chenxisd.shinyapps.io/pancaf/). **j** Relative expression of HAS1 between CAF^IF^ and CAF^TC^ by RT-PCR. **k** Relative expression of HAS1 between CAF^IF^ and CAF^TC^ by WB. **l**-**m** IHC analysis and graphically summarized. *P* = two-tailed t test. Graphical summary from IHC analysis of HAS1 stained stromal fibroblasts from tumor centers and invasion front of OSCC (*n* = 72). **n**-**q** Kaplan–Meier curves showing the correlation between HAS1⁺ CAF abundance and OS and DFS in 72 OSCC patients, based on CAF data from both the tumor center and invasion front. *P*-values were calculated using the log-rank test

To elucidate the molecular differences between CAF^TC^ and CAF^IF^, we conducted a gene transcriptome analysis. The results revealed significant gene-level differences among the four groups of CAFs (Fig. 4a). However, conventional CAF markers such as ACAT2, S100A4, FAP, PDGFRA, and PDGFRB did not show any significant variation (Fig. 4b). To identify characteristic genes of CAF^IF^, we performed a Venn diagram analysis and found several genes that were significantly upregulated in CAF^IF^, including ELN, HAS1, ICAM2, LSP1, CABP1, TMEM132b, RIPK4, MCAM, and ADGRG1 (Fig. 4c-d). Gene Ontology (GO) enrichment analysis indicated that CAF^IF^ were primarily enriched in pathways related to cell adhesion and extracellular matrix assembly (Fig. 4e). Notably, the log_2_FC value for HAS1 was the highest among these genes, prompting us to select HAS1 for further investigation.

To further characterized the subset of cells expressing HAS1 in human OSCC, we analyzed the correlation between HAS1 and differentially expressed genes across various tumor-associated cell populations in head and neck squamous cell carcinoma (HNSCC, *n* = 522, TCGA dataset, Firehose Legacy). Several markers of activated fibroblasts were positively correlated with HAS1 levels (Fig. 4f). Additionally, the estimate analysis indicated a significant relationship between HAS1 and the stromal components of OSCC (Fig. 4g). Further validation in the GSE103322 HNSCC single-cell database (comprising 18 samples, all of which are from OSCC cases) confirmed that HAS1 was predominantly expressed in fibroblasts within OSCC (Fig. 4h). Analyzing the pan-cancer CAF database, we found that HAS1 exhibited a closer association with the traditional inflammatory CAF (iCAF) subtype (Fig. 4i). We subsequently verified the expression of HAS1 in both CAF^IF^ and CAF^TC^, finding that HAS1 mRNA levels were indeed higher in CAF^IF^ compared to CAF^TC^ (Fig. 4j). Western blotting and immunofluorescence analysis further supported increased HAS1 expression in CAF^IF^ (Fig. 4k). Next, we conducted IHC staining for HAS1 in paraffin-embedded tissue sections from OSCC patients at our institution. Immunohistochemical scoring of CAF^TC^ and CAF^IF^ revealed significantly higher HAS1 expression in CAF^IF^ (*p* < 0.001) (Fig. 4l-m). Notably, patients exhibiting higher HAS1 expression specifically in the IF demonstrated poorer DFS (*p* = 0.042) (Fig. 4n-q). These findings suggested that CAF^IF^ of OSCC express elevated levels of HAS1, correlating with patient prognosis, and indicate that HAS1^+^ CAFs may play a role in the malignant biological processes of OSCC.

## HAS1 deficiency of CAF^IF^ impairs ECM reconstruction and inhibits tumor invasion

**Fig. 5:**
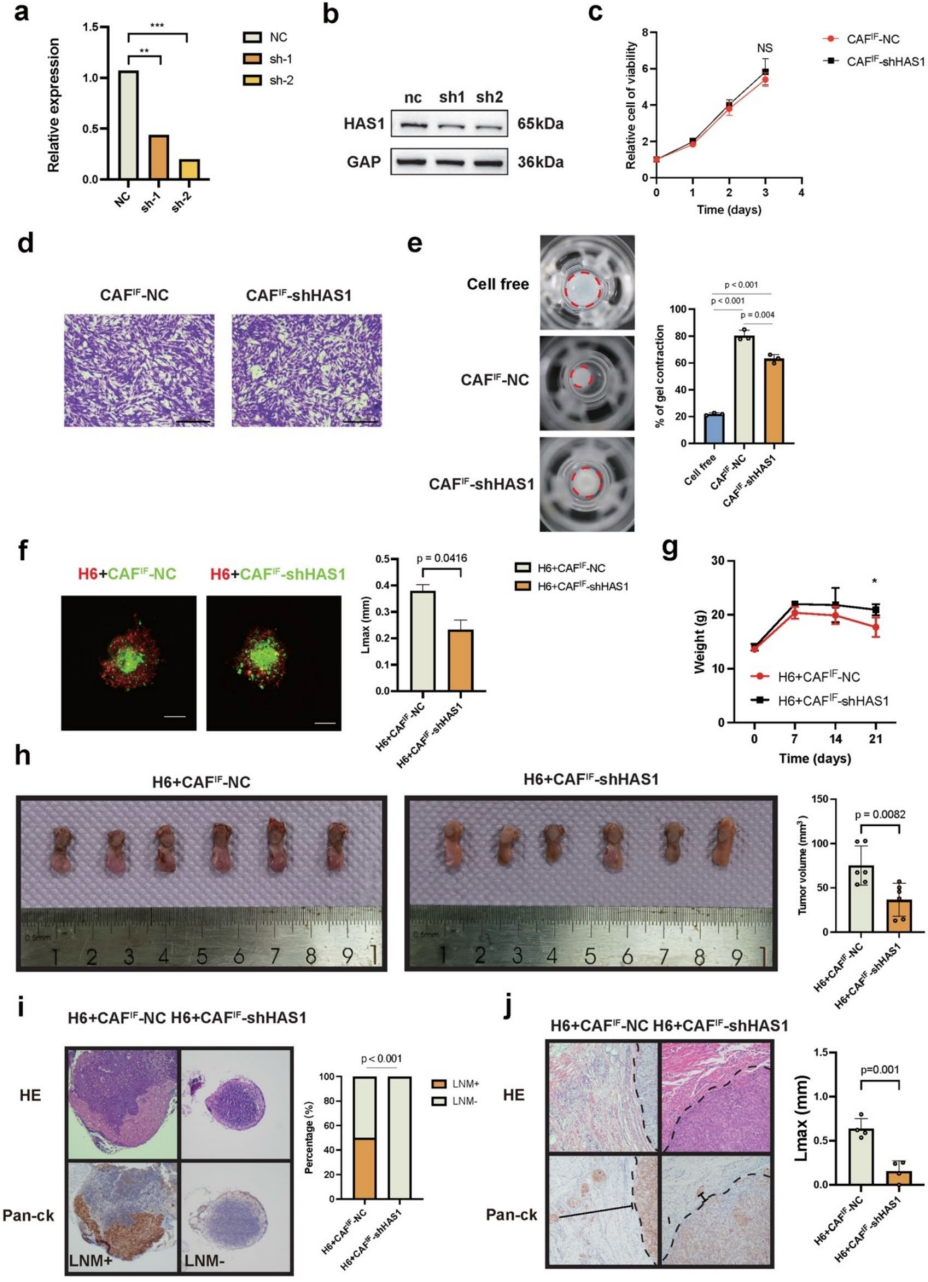
HAS1 deficiency impairs ECM reconstruction and tumor invasion. **a** Relative expression of HAS1 between CAF^IF^-NC, CAF^IF^—shHAS1-1 and CAF^IF^—shHAS1-2 by RT-PCR. **b** Relative expression of HAS1 between CAF^IF^-NC, CAF^IF^—shHAS1-1 and CAF^IF^—shHAS1-2 by WB. **c** The line graph shows the comparison of proliferation ability between CAF^IF^-NC and CAF^IF^-shHAS1 using CCK-8 assay. **d** The graph shows the comparison of migration between CAF^IF^-NC and CAF^IF^-shHAS1 using Transwell assays. Scale bar, 200 μm. **e** ECM remodeling was evaluated using a Matrix gel contraction assay (*n* = 3) among cell-free control, CAF^IF^-NC and CAF^IF^-shHAS1. *P* = Two-tailed t test. **f** Setup of heterotypic organoid formation from HN6-mcherry and CAF^IF^-NC-GFP and CAF.^IF^-shHAS1-GFP, cultured in Matrigel gels mixed with collagen I. The matrix invasion of CAF/HN6 heterotypic organoid was determined by estimating the maximal distance of invasion from the spheroid border (Lmax). *n* = 3/group, Two-tailed t test. Scale bars, 100 μm. **g** Graphical representation of changes in mouse weight following tumor implantation. Two-tailed t test (*n* = 6/group). **h** Graphical representation of tumor volume. Two-tailed t test (*n* = 6/group). **i** Graphical representation of LNM percentage. Pearson’s correlation test (*n* = 6/group). Scale bar: 200 μm. **j** Graphical representation of invasion distance in satellite lesions. Two-tailed t test (*n* = 6/group)

To assess the potential role of HAS1 expression in CAF^IF^-dependent OSCC tumor progression, we used lentiviral transduction to achieve a stable knockdown of HAS1 mRNA expression in CAF^IF^ (Fig. 5a). Western blotting analysis confirmed a reduction in HAS1 protein levels (Fig. 5b). We next evaluated the impact of HAS1 on the intrinsic functions of CAFs. First, we compared the proliferation rates of CAF^IF^-NC and CAF^IF^-shHAS1. CCK-8 assay results showed no significant difference in proliferation between CAF^IF^-NC and CAF^IF^-shHAS1 (*p* > 0.05, Fig. 5c). Transwell assays were also conducted to assess differences in migratory capacity, but results revealed no significant difference between CAF^IF^ and CAF^IF^-shHAS1 (Fig. 5d). Lastly, we assessed matrix remodeling capability using a gel contraction assay, and we found CAF^IF^-NC exhibited significantly greater matrix remodeling ability than CAF^IF^-shHAS1 (Fig. 5e).

We then explored the function of HAS1 expression on tumor cell regulation. Using an in vitro organoid model, we cocultured CAFs with HN6 cell spheroids to examine the effects on HN6 tumor cell invasion. Results indicated that CAF^IF^-NC substantially promoted the Lmax of HN6 compared to CAF^IF^-shHAS1, suggesting that HAS1 knockdown significantly reduces the pro-invasive capacity of HAS1-expressing CAFs in enhancing HN6 invasion (Fig. 5f). In vivo, compared to mice co-injected with HN6 and CAF^IF^-NC cells, those co-injected with CAF^IF^-shHAS1 cells exhibited significantly reduced tumor burden, local tissue invasion, and lymph node metastasis (Fig. 5h-j). These results indicated that HAS1^+^CAFs were involved in ECM reconstruction and tumor invasion.

## HAS1-high CAFs promoted OSCC progression by inducing EMT through ECM remodeling

**Fig. 6:**
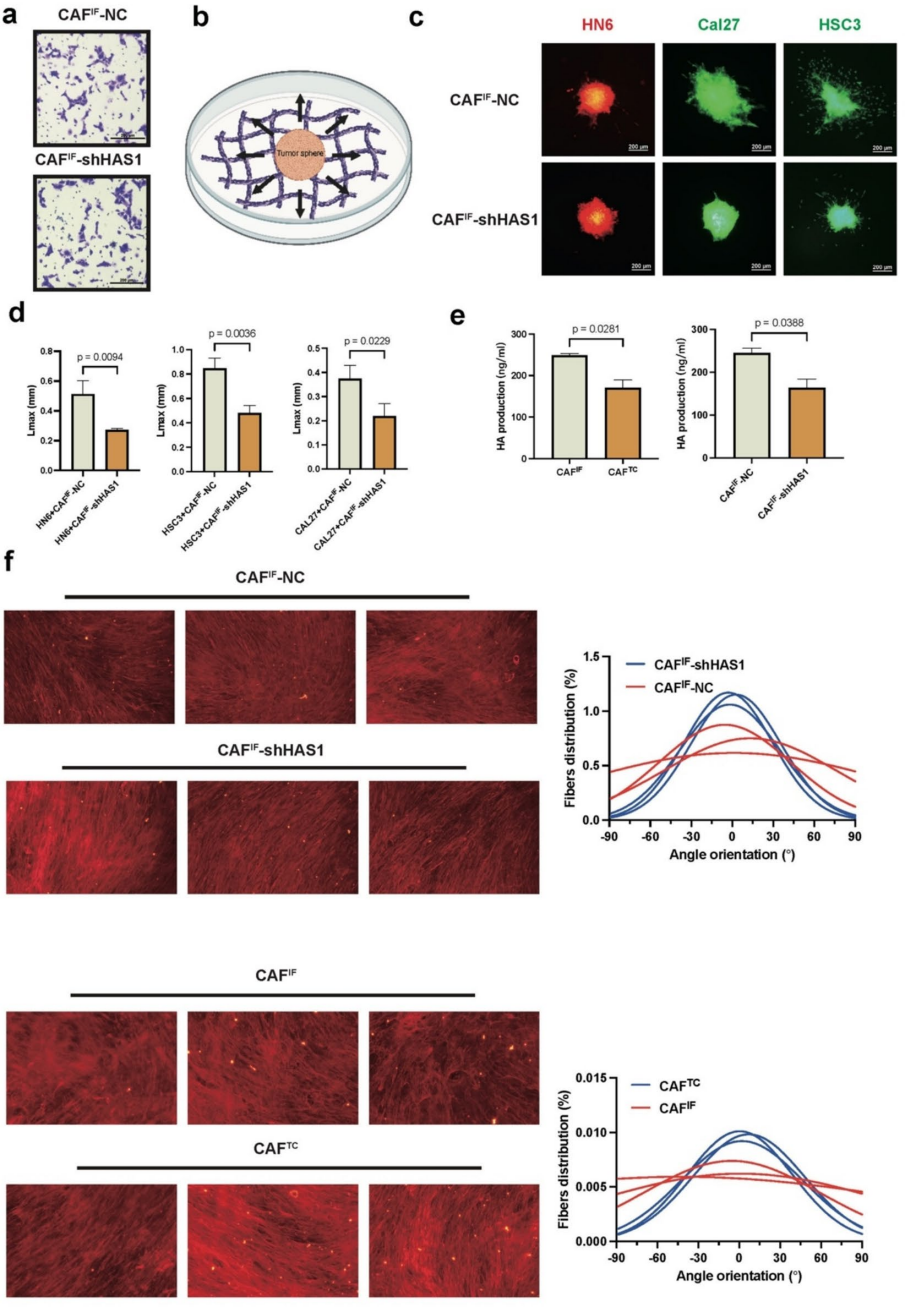
HAS1-dependent ECM remodeling promotes OSCC invasion. **a** The graph shows the comparison of HN6 migration ability induced by CAF^IF^-NC and CAF^IF^-shHAS1 using Transwell indirect co-culture (*n* = 3/group). **b** The schematic depicts a model showing how CAF-derived dECM influences tumor migration. **c** The graph shows the comparison of migration ability of HN6-mCherry, Cal27-GFP and HSC3-GFP induced by dECM generated from CAF^IF^-NC and CAF^IF^-shHAS1. Two-tailed t test (*n* = 3/group). **d** Quantification of OSCC cell migration induced by dECM generated from CAF^IF^-NC and CAF^IF^-shHAS1. The maximum migration distance (Lmax) of HN6, HSC3, and CAL27 cells was significantly reduced when cultured on dECM from CAF^IF^-shHAS1 compared to CAF^IF^-NC. Two-tailed t test (*n* = 3/group). **e** Relative expression of HA between CAF^IF^-NC and CAF^IF^-shHAS1, CAF^IF^ and CAF^TC^ by elisa. **f** Immunofluorescence imaging of dECM generated from CAF^IF^-NC and CAF^IF^-shHAS1, CAF^IF^ and CAF^TC^ (40x), and fiber orientation analysis using ImageJ

Since HAS1 serves as a marker for iCAFs, which are characterized by the secretion of cytokines and chemokines, it is also a key enzyme in the synthesis of HA, a critical component of the ECM [15]. Then, we investigated the role of CAF^IF^ in paracrine signaling and ECM remodeling during the regulation of OSCC invasion. Indirect co-culture experiments were first conducted to assess the effects of cytokines and chemokines secreted by the CAF^IF^-NC and CAF^IF^-shHAS1 groups on HN6 cell migration, which revealed no significant differences (Fig. 6a). HAS1-high CAFs did not regulate OSCC invasion via paracrine secretion of cytokines and chemokines. Thus, we hypothesized that HAS1-high CAFs may influence OSCC invasion through extracellular matrix remodeling. To test this hypothesis, we evaluated the impact of ECM produced by CAF^IF^-NC and CAF^IF^-shHAS1 groups on HN6, HSC3, and CAL27 cell invasion. By measuring the maximum invasion distance of HN6, HSC3, and CAL27 tumor spheroids on dECM derived from CAF^IF^-NC and CAF^IF^-shHAS1, we observed that knockdown of HAS1 in CAF^IF^ significantly impaired the pro-invasive capacity of CAF^IF^-derived ECM (*p* < 0.05) (Fig. 6b-c). These findings suggested that the pro-invasive effects of HAS1-high CAF^IF^ may primarily arise through ECM remodeling. To further explore the potential mechanism, ELISA showed significantly higher HA production in CAF^IF^ than in CAF^TC^ (*p* = 0.002). CAF^IF^-shHAS1 exhibited reduced HA levels compared to CAF^IF^-NC (*p* = 0.039), suggesting that HAS1 mediates HA production in CAF^IF^ (Fig. 6e). Compared to CAF^TC^, CAF^IF^ exhibited a more chaotic arrangement of collagen fibers, indicating a disorganized and isotropic ECM structure.Interestingly, CAF^IF^-shHAS1 exhibited a more organized arrangement compared to CAF^IF^-NC, as evidenced by the narrower distribution of fiber orientation angles (Fig. 6f).

**Fig. 7:**
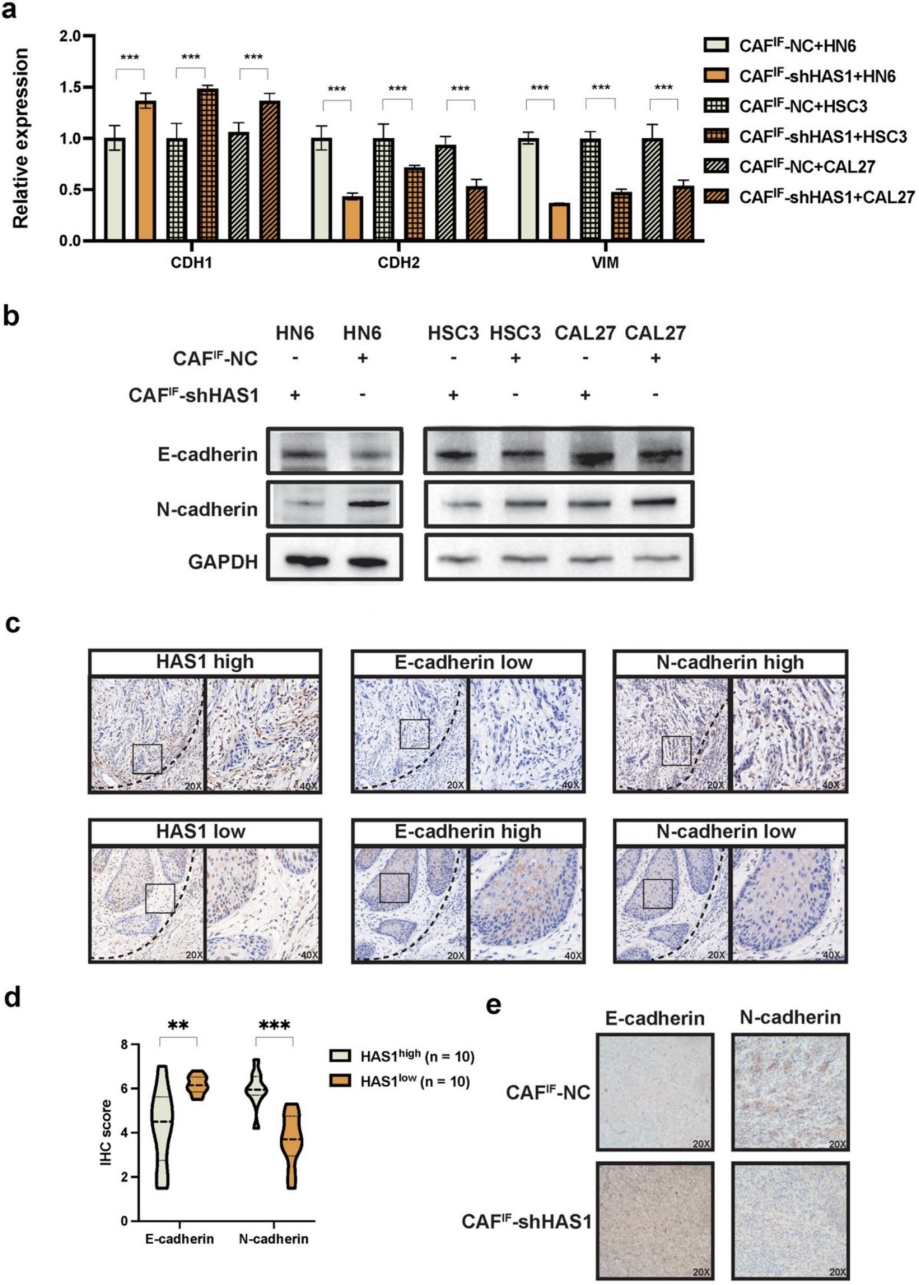
HAS1 knockdown in CAF^IF^ suppresses EMT in OSCC cells through ECM remodeling**. a** Graph showing the relative expression of EMT in HN6, Cal27 and HSC3 induced by dECM generated from CAF^IF^-NC and CAF^IF^-shHAS1, as assessed by RT-PCR. **b** Representative Western blot showing protein levels of E-cadherin and N-cadherin in the same cells cultured on dECM from CAF^IF^-NC or CAF^IF^-shHAS1. GAPDH was used as a loading control. **c** Representative immunohistochemical staining of serial sections from OSCC patient specimens showing the spatial association between HAS1 expression and EMT markers. Tumor epithelial cells at the invasive front with high HAS1 expression exhibited reduced E-cadherin and elevated N-cadherin levels, while HAS1-low regions showed the opposite pattern. Left panels: 20 × magnification; right panels: 40 × magnification. **d** Quantification of E-cadherin and N-cadherin immunohistochemical staining in OSCC tissues (*n* = 10 per group), demonstrating significant correlation with HAS1 expression levels. **e** Representative IHC images of orthotopic xenograft tumors derived from co-injection of HN6 cells with either CAF^IF^-NC or CAF^IF^-shHAS1. Tumors in the CAF^IF^-NC group exhibited EMT-like marker expression, whereas tumors in the CAF^IF^-shHAS1 group showed reversed EMT phenotypes. All images were acquired at 20 × magnification

To clarify the mechanism by which HAS1-high CAFs remodel the ECM to regulate OSCC cell invasion, we compared the effects of dECM from CAF^IF^-NC and CAF^IF^-shHAS1 on the EMT of HN6, HSC3, and CAL27 cells. qPCR analysis revealed that dECM from CAF^IF^-NC significantly upregulated EMT-related gene expression in HN6, HSC3, and CAL27 cells (*p* < 0.001). Consistently, Western blotting confirmed increased levels of EMT markers in HN6, HSC3, and CAL27 cells cultured on CAF^IF^-NC dECM (Fig. 7a–b). To further validate these findings in clinical specimens and animal models, we examined EMT marker expression in OSCC tissues and mouse orthotopic xenografts. Immunohistochemical staining of serial OSCC sections revealed that IF with high HAS1 expression showed markedly lower E-cadherin and higher N-cadherin levels in tumor epithelial cells, compared to HAS1-low regions (Fig. 7c). IHC scoring confirmed that HAS1-high areas were significantly associated with reduced E-cadherin (*p* < 0.01) and increased N-cadherin expression (*p* < 0.01, Fig. 7d). In the orthotopic xenograft model, tumors derived from CAF^IF^-NC and HN6 co-injection displayed decreased E-cadherin and increased N-cadherin expression, while those in the CAF^IF^-shHAS1 group showed a reversal of this EMT phenotype (Fig. 7e). These findings from both clinical samples and in vivo models consistently suggest an association between HAS1^+^ CAF^IF^ and EMT phenotypes in OSCC cells.

## Discussion

**Fig. 8:**
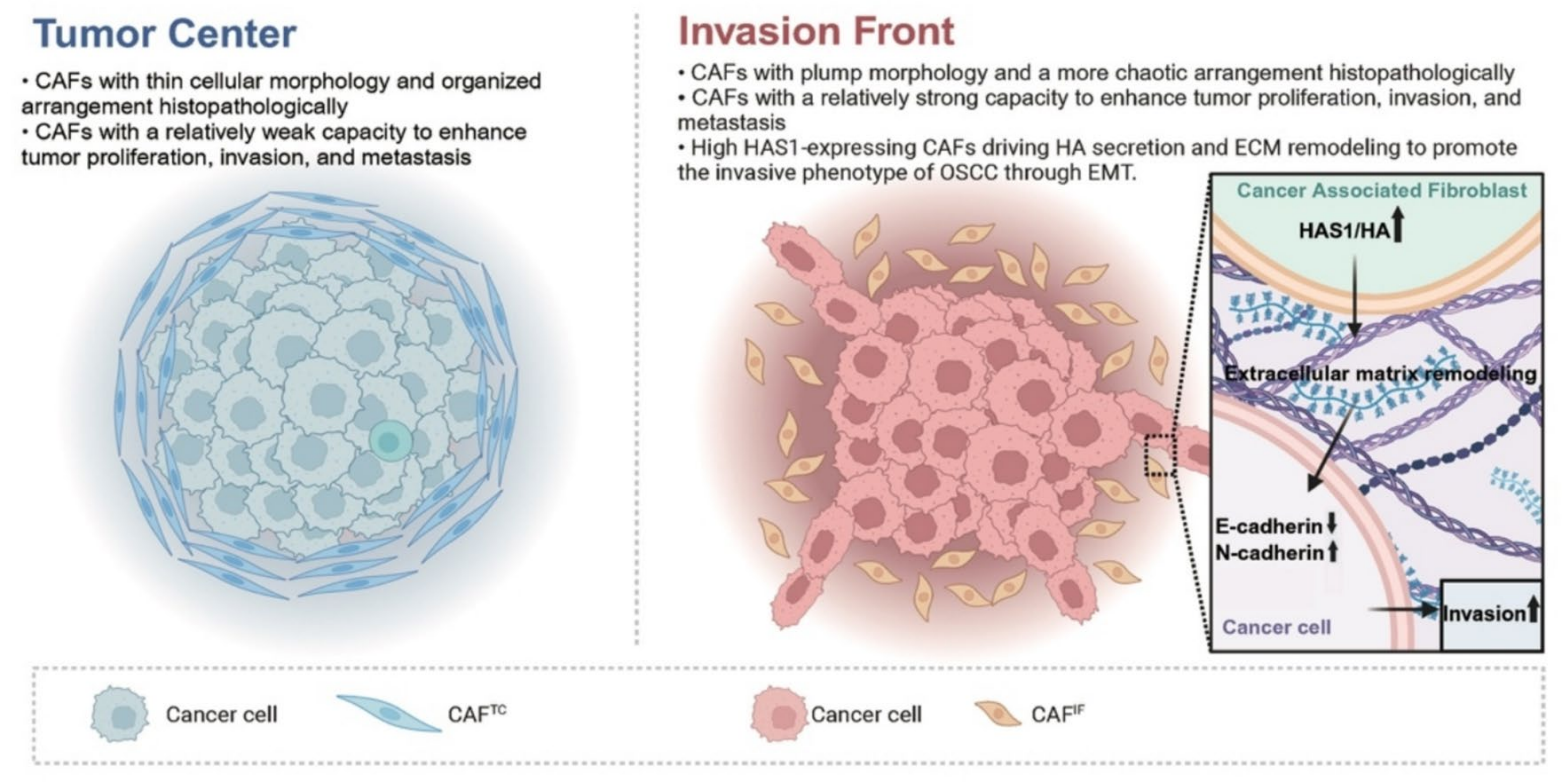
The proposed working model depicts the morphological differences between CAFs in the tumor center and those at the invasion front, and the mechanism by which invasion front CAFs, through high expression of HAS1, drive hyaluronic acid (HA) secretion and ECM remodeling, promoting the EMT and invasive phenotype of OSCC

Currently, the morphological and molecular characteristics of tumor cells at the IF in OSCC are relatively well understood. However, CAFs, which are complex in function and diverse in subtypes, play a critical role in regulating tumor progression. The clinical significance and molecular differences between CAF^TC^ and CAF^IF^ still remain unclear. Our study revealed significant morphological differences between these CAFs and highlighted the higher clinical relevance of CAF^IF^. Specifically, research focusing on CAF^IF^ demonstrated their more pronounced role in regulating OSCC progression. In particular, CAF^IF^ promote the invasive phenotype of OSCC by highly expressing HAS1, thereby maintaining HA secretion and remodeling ECM (Fig. 8).

The characteristics of the IF have long drawn significant attention. Carnielli et al. conducted a microdissection on OSCC paraffin sections to collect stromal tissues from both the TC and IF, followed by proteomics analysis [18]. They found increased expression of COL6A1, ITGAV, and myoglobin in the stromal components at the OSCC IF. However, the functional roles of these stromal components in regulating OSCC have not been further explored. Similarly, Lu et al., through immunohistochemistry, studied TGF-β expression in OSCC and found significantly higher expression of TGF-β in CAFs at the IF compared to those at the TC. However, the specific implications of this finding were not further investigated [22].

Due to the use of paraffin sections, where cells are generally non-viable, studying the regulatory roles of CAFs on tumor cells is challenging. However, isolating primary CAFs from tumor patients holds excellent significance for functional research. Zhang et al. isolated CAFs and normal fibroblasts from OSCC patients and discovered that CAFs promote OSCC proliferation by highly expressing ITGB2, which activates the PI3K/AKT/mTOR axis [23]. Similarly, Ding et al. extracted CAFs from OSCC patients classified as WPOI1-3 and WPOI4-5, finding that WPOI4-5 CAFs were characterized by high OXTR expression, promoting OSCC invasion [19]. Since the IF in OSCC is located at the leading edge of tumor invasion, approximately 1 mm wide, isolating primary cells from this region is challenging. We utilized a 16G biopsy needle with an internal diameter of about 1 mm, similar to the width of the IF, to extract cells that sufficiently reflect the molecular and functional characteristics of CAFs in this region. Additionally, the remaining tumor tissues were prepared as H&E sections to observe the sampling site, ensuring that samples were exclusively from the IF. This method holds promise for addressing the challenges of in-depth research on the IF of malignant tumors.

In breast cancer, high HAS1 expression in stromal cells is significantly associated with lymph node metastasis, tumor size, and poor prognosis [24], findings similar to ours. However, our study revealed that high HAS1 expression in CAF^IF^, rather than in CAF^TC^, correlates with lower DFS in patients. In pancreatic cancer, HAS1 is identified as one of the markers for iCAFs, which regulate tumor progression primarily through the secretion of growth and inflammatory factors [25]. However, our study showed that CAFs with high HAS1 expression promote OSCC invasion not through growth or inflammatory factor secretion but via ECM remodeling.

CAF^IF^ exhibited strong capabilities in ECM remodeling, HA secretion, and altering collagen I fiber alignment. As a critical ECM component, HA is closely associated with tumor progression. Single-cell sequencing and ligand-receptor relationship analysis in liver cancer have shown significant interactions between HA secreted by myCAFs and tumor receptors such as CD44 and HMMR [26]. High HA content in the stroma is significantly associated with lymph node metastasis and tumor size in breast cancer. Furthermore, the study revealed that high HA content in the stroma transforms the matrix into a mucin-like state [27]. Our findings also demonstrated distinct characteristics of CAF^TC^ and CAF^IF^. CAF^TC^, with low HAS1 expression, are characterized by slender cells arranged in an orderly manner, while CAF^IF^, with high HAS1 expression, exhibit plump cells arranged chaotically.

In conclusion, our study highlighted significant morphological differences between CAF^TC^ and CAF^IF^, with CAF^IF^ showing higher clinical relevance. Specifically, our findings demonstrated that CAF^IF^ play a more prominent role in regulating OSCC progression, mainly through the high expression of HAS1, which drives HA secretion and ECM remodeling, promoting the EMT and invasive phenotype of OSCC.

## Supplementary Information

Supplementary Material 1.

## Data Availability

The data supporting the findings of this study are available from the corresponding author upon reasonable request.

## Abbreviations

TC: Tumor Center
IF: Invasion Front
OSCC: Oral Squamous Cell Carcinoma
CAF: Cancer-Associated Fibroblast
H&E: Hematoxylin and Eosin
CAF^IF^: CAFs from the Invasion Front
CAF^TC^: CAFs from the Tumor Center
HAS1: Hyaluronan Synthase 1
HA: Hyaluronic Acid
TNM: Tumor-Node-Metastasis
WHO: World Health Organization
UICC: Union for International Cancer Control
FBS: Fetal Bovine Serum
OS: Overall Survival
DFS: Disease-Free Survival
HR: Hazard Ratio
CI: Confidence Interval
WPOI: Worth pattern of invasion
