# Supplementary material for: HAS1high cancer associated fibroblasts located at the tumor invasion front zone promote oral squamous cell carcinoma invasion via ECM remodeling
Source: J Exp Clin Cancer Res. 2025 Aug 14;44:238. doi: 10.1186/s13046-025-03493-6 (PMC12351772; doi:10.1186/s13046-025-03493-6)
Supplement: Supplementary file 1 — Supplementary Material 1. [file 13046_2025_3493_MOESM1_ESM.docx]

**Construction of RNA sequencing libraries and sequencing**

Total RNA was extracted from the samples by Trizol reagent (Invitrogen) separately. The RNA quality was checked by Agilent 2200 and kept at −80°C. The RNA with RIN (RNA integrity number) > 7.0 is acceptable for cDNA library construction. The cDNA libraries were constructed for each RNA sample using the TruSeq Stranded mRNA Library Prep Kit (Illumina, Inc.) according to the manufacturer’s instructions. Generally, the protocol consists of the following steps: Poly-A containing mRNA was purified from 1ug total RNA using oligo(dT) magnetic beads and fragmented into 200-600 bp using divalent cations at 85℃ for 6 min. The cleaved RNA fragments were used for first- and second-strand complementary DNA (cDNA) synthesis. dUTP mix was used for second-strand cDNA synthesis, which allows for the removal of the second strand. The cDNA fragments were end repaired, A-tailed and ligated with indexed adapters. The ligated cDNA products were purified and treated with uracil DNA glycosylase to remove the second-strand cDNA. Purified first-strand cDNA was enriched by PCR to create the cDNA libraries. The libraries were quality controlled with Agilent 2200 and sequenced by NovaSeq 6000 on a 150 bp paired-end run.

**RNA sequencing Mapping:**

Before read mapping, clean reads were obtained from the raw reads by removing the adaptor sequences and low-quality reads. The clean reads were then aligned to human genome (GRCh38) using the Hisat2. HTseq was used to get gene counts and FPKM method was used to determine the gene expression.

**Dif-Gene Analysis:**

We applied EB-Seq algorithm to filter the differentially expressed genes, after the significant analysis, Pvalue and FDR analysis under the following criteria: i) Fold Change>2 or <0.5; ii) ,FDR<0.05.

**Go Analysis:**

Gene ontology (GO) analysis was performed to facilitate elucidating the biological implications of the differentially expressed genes in the experiment. We downloaded the GO annotations from NCBI (http://www.ncbi.nlm.nih.gov/), UniProt (http://www.uniprot.org/) and the Gene Ontology (http://www.geneontology.org/). Fisher’s exact test was applied to identify the significant GO categories (P-value < 0.05).
